# A Case Report of Pheochromocytoma Presenting With a Tongue Sign Indicative of Blood Deficiency

**DOI:** 10.7759/cureus.79880

**Published:** 2025-03-01

**Authors:** Rinne Shimizu, Akihiro Asakawa, Hajime Suzuki, Marie Amitani, Keiko Kawano, Haruka Amitani, Marie Hirahara, Keisuke Matsushita, Yousuke Horikiri, Oki Toshimichi, Yoshihiko Nishio, Koji Yonemori

**Affiliations:** 1 Department of Psychosomatic Internal Medicine, Kagoshima University Hospital, Kagoshima, JPN; 2 Kampo Medical Center, Kagoshima University Hospital, Kagoshima, JPN; 3 Department of General Medicine, Ryokusenkai Yonemori Hospital, Kagoshima, JPN; 4 Department of Psychosomatic Internal Medicine, Kagoshima University Graduate School of Medical and Dental Sciences, Kagoshima, JPN; 5 Department of Oral and Maxillofacial Surgery, Kagoshima University Graduate School of Medical and Dental Sciences, Kagoshima, JPN; 6 Department of Community-Based Medicine, Kagoshima University Graduate School of Medical and Dental Sciences, Kagoshima, JPN; 7 Department of Diabetes and Endocrine Medicine, Kagoshima University Hospital, Kagoshima, JPN; 8 Department of Reproductive Health Nursing, Faculty of Medicine, School of Health Science, Kagoshima University, Kagoshima, JPN; 9 Department of Diabetes and Endocrine Medicine, Kagoshima University Graduate School of Medical and Dental Sciences, Kagoshima, JPN; 10 Orthopedic Surgery, Department of Orthopedic, Yonemori Hospital, Social Medical Corporation Ryokusenkai, Kagoshima, JPN

**Keywords:** blood deficiency, pheochromocytoma, saikokaryukotsuboreito (srb), sympathetic hypertonia, tongue sign

## Abstract

Pheochromocytoma is a rare neuroendocrine tumor presenting with varied clinical symptoms. We present a unique case of pheochromocytoma in a 46-year-old male who developed paroxysmal palpitations, headaches, and elevated blood pressure. Notably, his tongue showed atypical signs of blood deficiency (pale white, thin, and emaciated appearance), which we attributed to peripheral vasoconstriction from sympathetic hyperactivity. His pulse and abdominal findings aligned with qi counterflow in Kampo medicine (traditional Japanese herbal medicine).

Despite treatment with Saikokaryukotsuboreito, a traditional herbal formula prescribed to address qi counterflow, only partial improvement of neuropsychiatric symptoms was observed. Further evaluation by an endocrinology specialist confirmed the diagnosis of pheochromocytoma through advanced diagnostic modalities, including scintigraphy. Pheochromocytoma is known to induce sympathetic hyperactivity via excessive catecholamine production, resulting in peripheral vasoconstriction and compromised blood flow, particularly in distal areas such as the tongue.

This case illustrates that sympathetic hyperactivity can produce tongue signs mimicking blood deficiency patterns. Recognition of this discrepancy between tongue signs and other clinical findings prompted further investigation, leading to the diagnosis of pheochromocytoma through modern diagnostic methods. Our experience demonstrates how the integration of Kampo and Western diagnostic approaches enables accurate diagnosis of underlying conditions.

## Introduction

Pheochromocytoma is a neuroendocrine tumor characterized by the overproduction of catecholamines, leading to sympathetic hypertonia. This condition can cause widespread constriction of peripheral blood vessels, eliciting a range of symptoms including hypertension, headaches, palpitations, sweating, and anxiety [1]. It has been reported that approximately 0.1%-0.2% of adult hypertensive patients are diagnosed with pheochromocytoma, highlighting its rarity and clinical significance [2]. Although pheochromocytoma can manifest atypical symptoms, potentially delaying recognition and complicating early diagnosis [3].

Kampo medicine has proven effective for various symptoms that are challenging to diagnose and treat with Western medicine alone. In Japan, Kampo medicine has been integrated into the national medical system, with treatments covered by health insurance. While Western medicine focuses on disease-specific diagnosis and treatment, Kampo medicine employs a holistic approach that considers the patient's overall physical condition and symptoms. Many patients who are resistant to Western medical treatments seek specialized Kampo medical institutions, hoping to find relief for their symptoms. This demonstrates how Kampo and Western medicine can function as complementary approaches to healthcare [4-7]. Consequently, physicians practicing Kampo medicine must develop expertise in both Kampo diagnostic methods and Western medical diagnosis and treatment protocols [8].

In the present case, the patient exhibited signs of qi counterflow, as described in Kampo medicine [9]. Qi counterflow is a term in Kampo medicine that describes the reversal of the normal downward flow of qi, which in Western medicine closely correlates with autonomic nervous system function [10]. The patient's neuropsychiatric symptoms improved with the administration of Saikokaryukotsuboreito (SRB). Intriguingly, despite other indicators, the patient also exhibited atypical tongue signs that were inconsistent with the clinical presentation. To the best of our knowledge, there are no existing reports detailing tongue signs in patients with a diagnosis of pheochromocytoma. Therefore, we present this case along with a review of the relevant literature.

## Case presentation

**Figure 1:**
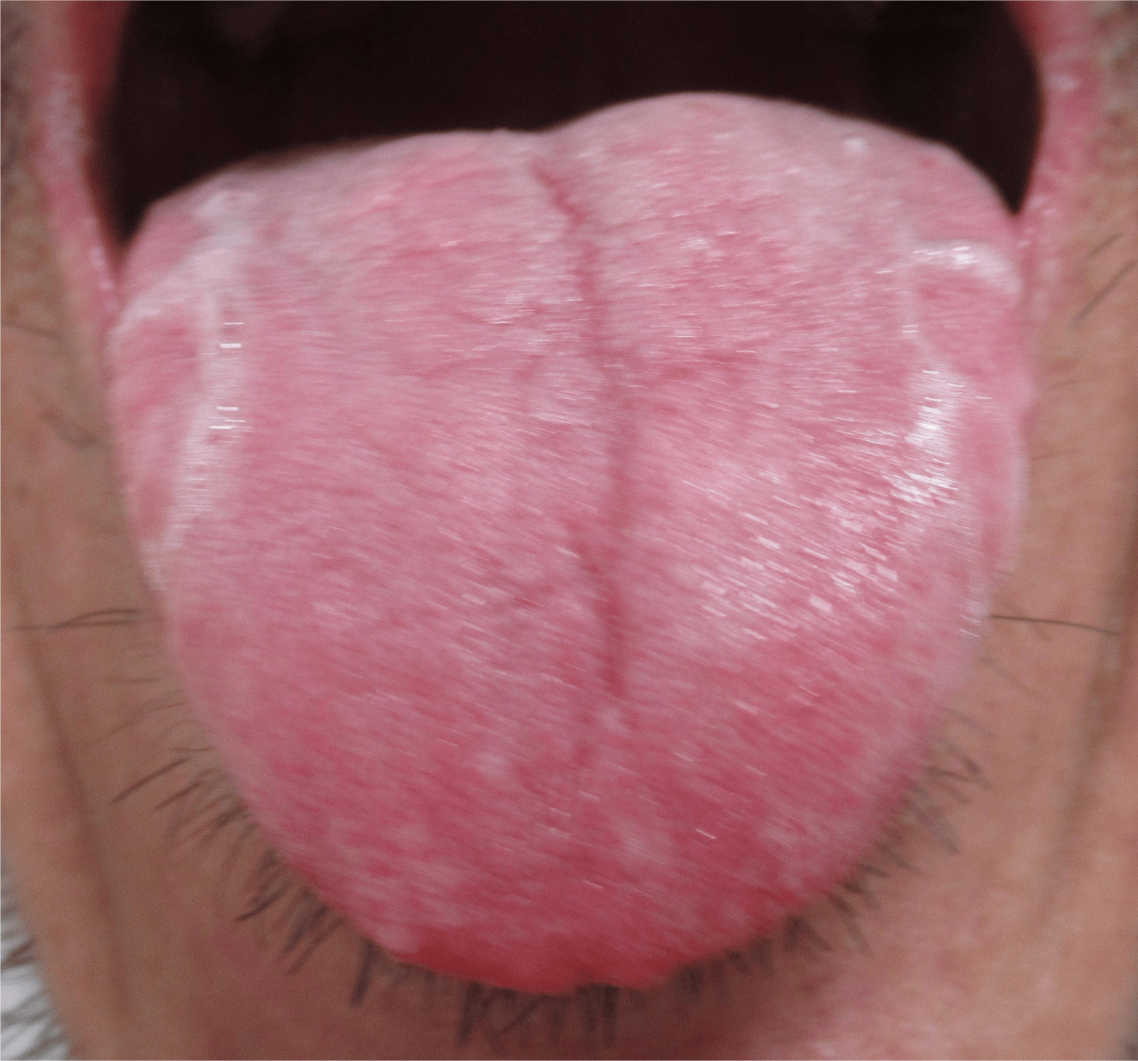
Tongue signs in April. The tongue appeared pale red (indicating a normal tongue color) with a slightly reddish tip, a thick texture, and tooth marks. The tongue also had a faint white coating, and no sublingual venous engorgement was noted. These findings, particularly the thick tongue with tooth marks, suggested qi deficiency and were consistent with the initial HST pattern. No signs of blood deficiency were observed at this stage. HST, hangeshashinto

In April 2022, the patient presented with a primary complaint of epigastric fullness that had persisted for approximately two years. His initial signs included epigastric discomfort and resistance, consistent with the hangeshashinto (HST) pattern. The tongue appeared pale red with a reddish tip, thickened texture, and tooth marks. The tongue also had a faint white coating, and no sublingual venous engorgement was noted (Figure 1). The eyes showed no pale conjunctiva, and the nails showed no pallor, vertical ridges, or brittleness. The Western medical evaluation revealed no evidence of anemia with a normal complete blood count.

The patient was treated with HST at a dose of 7.5 g/day for four weeks. Treatment was discontinued after his symptoms improved. A follow-up examination four weeks later showed a slight decrease in redness at the tip of the tongue, but no other notable changes were observed. The patient did not wish to undergo further treatment at this time, leading to a temporary cessation of consultations. In early July 2022, the patient returned with new-onset paroxysmal palpitations and headaches. Concurrently, he also reported an increase in blood pressure. He expressed a desire to explore Kampo's medical treatments for these symptoms. Specific medical findings from the patient's first and second visits, along with laboratory results from the second visit, are detailed in Table 1.

**Table 1:** Progression of main complaints and Kampo medicine findings. Clinical findings during Visit 1 and Visit 2 are summarized, including the patient's main complaints, blood pressure, subjective findings, and Kampo medicine-based evaluations (inspection, tongue inspection, pulse examination, and abdominal examination). Specific differences in tongue appearance, pulse characteristics, and abdominal strength are highlighted between the two visits.

		Visit 1	Visit 2
Main complaint		Epigastric fullness	Paroxysmal palpitations and headaches
Blood pressure		149/107 mmHg	155/117 mmHg
Kampo medicine findings	Subjective findings	・Mild fatigue	・Sleep disturbances・Agitation, irritability・Cold extremities
	Inspection	・Muscular and toned・Sharp-eyed	・Muscular and toned・More sharp-eyed・Facial flushing
	Tongue inspection	・Pale red overall with reddish tip・Thick with tooth marks・Tongue coating: slight white	・Pale white overall・Thin and emaciated with more tooth marks・Tongue coating: slight white
	Pulse examination	・Floating pulse ・String-like pulse	・Rapid pulse・Floating pulse・String-like pulse
	Abdominal examination	・Abdominal strength = 3-4/5・Epigastric stuffiness and resistance・Rumbling in the hypochondrium	・Abdominal strength = 3-4/5・Hypochondriac discomfort and distension・Brisk pulsation in the supra-umbilical region

**Figure 2:**
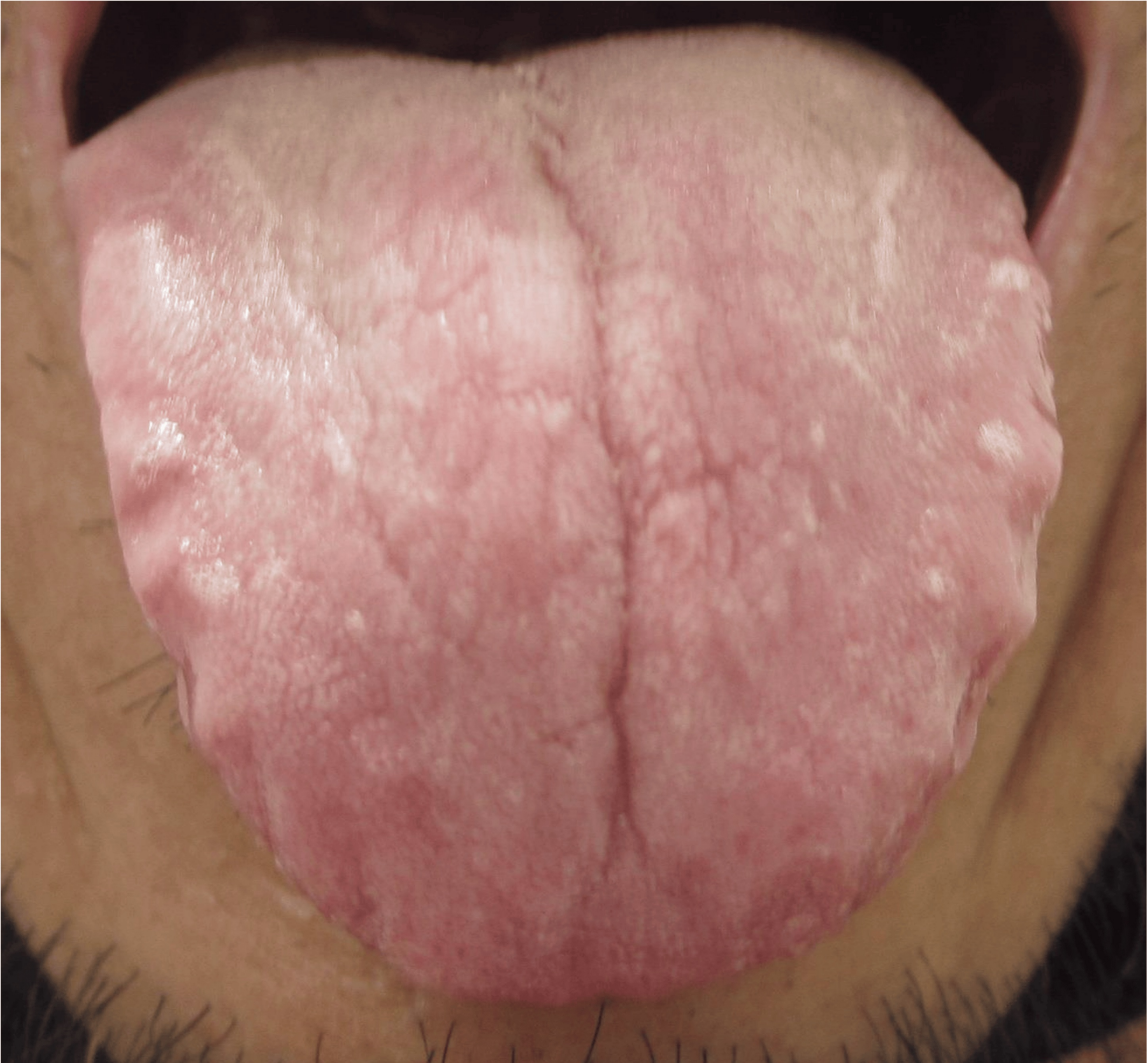
Tongue signs in July. Sequential changes in tongue appearance over a three-month period. Initially, the tongue was pale red and thick, with visible tooth marks. By the follow-up, the tongue had turned pale white (indicating a loss of normal color), become thin, and appeared emaciated, showing signs consistent with blood deficiency. This rapid progression to blood deficiency-like signs, particularly the progression to a pale white and thin tongue, reflected peripheral vasoconstriction due to sympathetic hyperactivity, which led to further investigation for pheochromocytoma.

At the second visit, the patient reported paroxysmal headaches and palpitations, accompanied by significant anxiety. Regarding sleep disturbances, the patient stated, "I was wide awake all night and couldn't fall asleep." Kampo's findings included facial flushing, brisk pulsation in the supra-umbilical region, cold extremities, and hypochondriac discomfort. These symptoms and findings suggested *qi counterflow* as the primary Kampo pattern. To address this, SRB was prescribed at a dose of 7.5 g/day. In contrast, the tongue's appearance had significantly changed over three months; it was pale white, thin, and emaciated, indicating blood deficiency (Figure 2), which was markedly different from the earlier pale red and thick tongue. Given the patient's persistently elevated blood pressure, screening tests for secondary hypertension were performed. 

**Figure 3:**
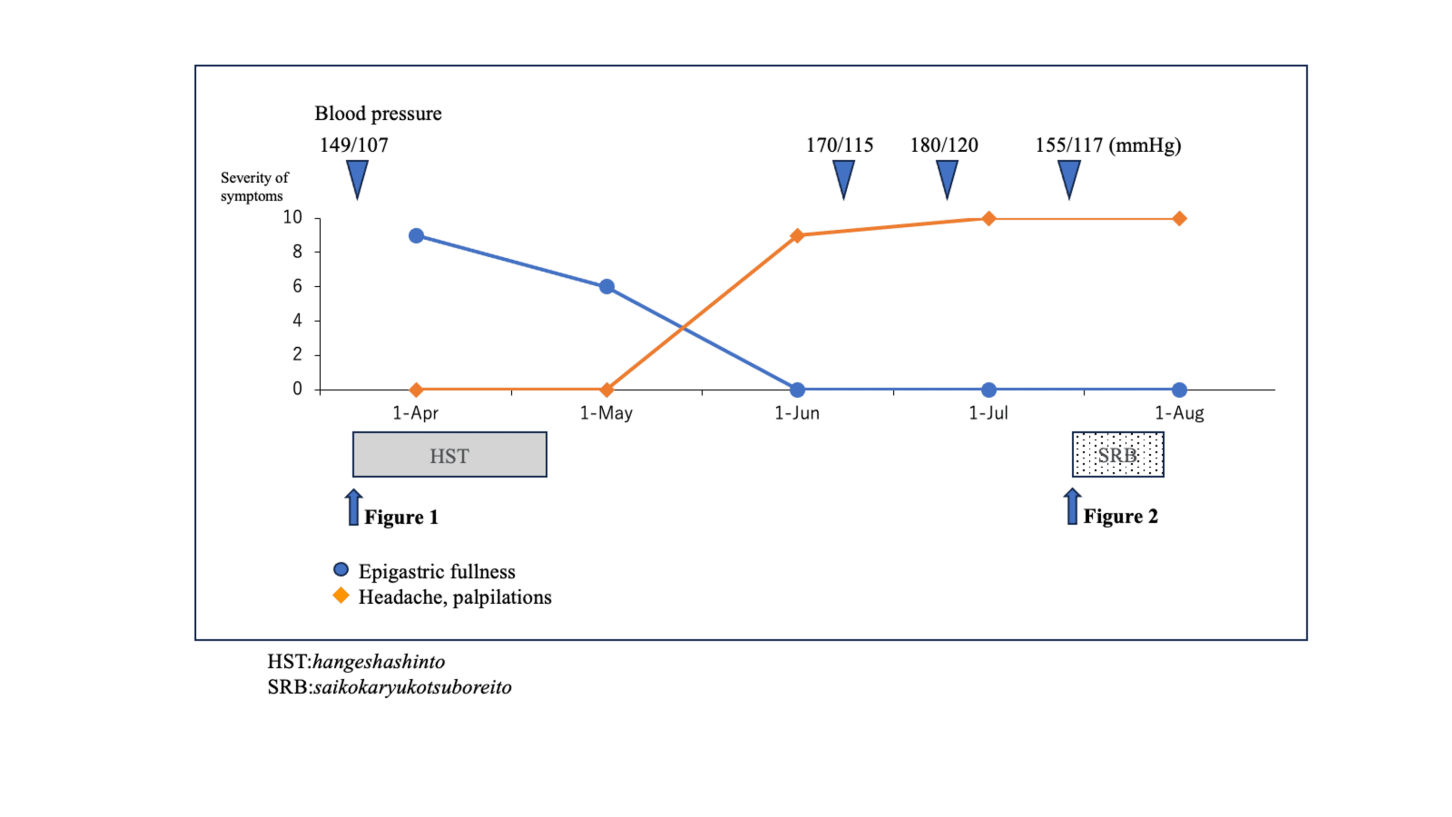
Symptom progression chart. The timeline illustrates the progression of the patient's symptoms, including paroxysmal palpitations, headaches, and elevated blood pressure, before and during Kampo treatment with Saikokaryukotsuboreito (SRB). An evaluation of the subjective symptoms was carried out by a numerical rating scale. Despite partial alleviation of neuropsychiatric symptoms, including sleep disturbances, the patient's tongue signs indicative of blood deficiency persisted. These findings ultimately led to further evaluation and the eventual diagnosis of pheochromocytoma.

At follow-up, the patient reported improved sleep duration from 3 to 6 hours per night with SRB treatment. His subjective anxiety levels had decreased, allowing him to feel more composed during daily activities. Despite this partial improvement in psychiatric symptoms, headache and palpitations persisted. Blood pressure also remained high throughout the treatment period (Figure 3).

Thyroid and adrenal cortical functions were normal, and primary aldosteronism was ruled out (Table 2). Initial endocrine workup revealed elevated urinary metanephrine and normetanephrine (Table 3). 

**Table 2:** Biochemical test. The results of biochemical tests are presented in the table. The parameters include liver enzymes (AST, ALT, LDH), creatine kinase (CK), kidney function markers (BUN, Cr), glucose (Glu), and electrolytes (Na, K, Cl). Reference ranges are provided for comparison. All values are within normal ranges, and no abnormalities were detected. AST, aspartate aminotransferase; ALT, alanine aminotransferase; LDH, lactate dehydrogenase; CK, creatine kinase; BUN, blood urea nitrogen; Cr, creatinine; Na, sodium; K, potassium; Cl, chloride; Glu, glucose

Biochemistry	Value	Unit	Reference range
AST	21	U/L	13-30
ALT	31	U/L	10-42
LDH	187	U/L	124-222
CK	98	U/L	59-248
BUN	9.0	mg/dL	8-20
Cr	0.69	mg/dL	0.65-1.07
Na	141	mEq/L	138-145
K	4.3	mEq/L	3.6-4.8
Cl	104	mEq/L	101-108
Glu	138	mg/dL	73-109

**Table 3:** Endocrinological test. The results of endocrinological tests are presented in the table. TSH, thyroid-stimulating hormone; F-T4, free thyroxine; ACTH, adrenocorticotropic hormone; PRA, plasma renin activity; PAC, plasma aldosterone concentration; Uro-Metanephrine, urinary metanephrine; Uro-Normetanephrine, urinary normetanephrine

Endocrinology	Value	Unit	Reference range
TSH	0.92	mIU/mL	0.61-4.23
F-T4	0.82	ng-dL	0.70-1.48
ACTH	41.6	pg/mL	7.2-63.3
Cortisol	12.0	mg/dL	4.5-21.1
PRA	3.2	ng/mL/hour	0.2-2.3
PAC	63.5	pg/mL	4.0-82.1
Uro-Metanephrine	6,840	ng/mg・Cr	<500
Uro-Normetanephrine	3,250	ng/mg・Cr	<500

**Figure 4:**
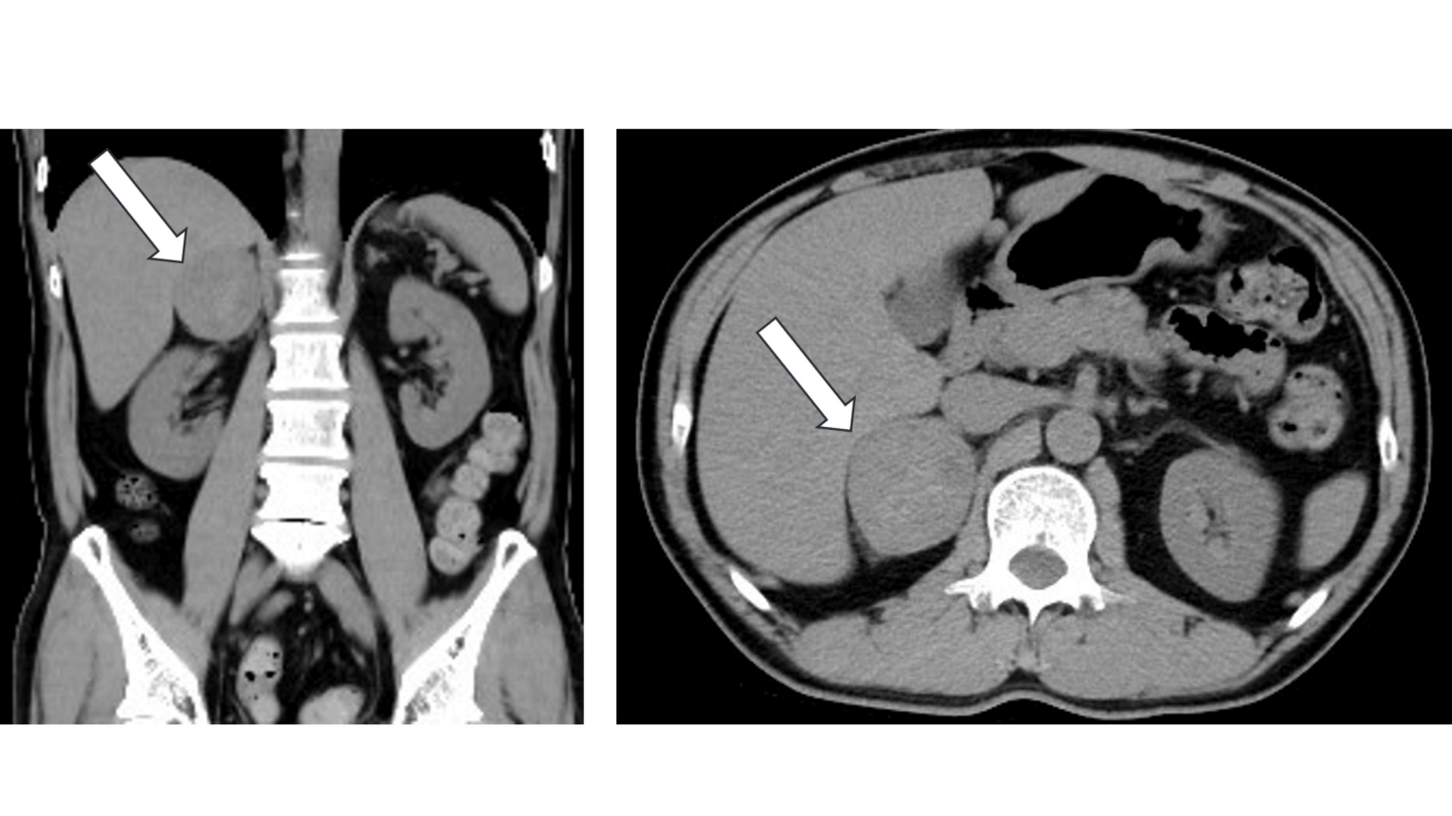
Simple computed tomography (CT) scan of the abdomen. The CT scan reveals a well-defined, borderline, slightly heterogeneous mass lesion measuring approximately 5 cm in diameter in the right adrenal gland. This finding, combined with elevated urinary metanephrine and normetanephrine, is consistent with the diagnosis of pheochromocytoma.

CT scan showed a 5-cm well-defined mass in the right adrenal gland (Figure 4).

Upon suspicion of pheochromocytoma, Kampo medicine was discontinued, and the patient was referred to an endocrinology specialist. Subsequent 123I-MIBG scintigraphy demonstrated intense radiotracer uptake in the right adrenal mass, confirming pheochromocytoma. The patient did not resume Kampo treatment thereafter.

## Discussion

In this case, a 46-year-old male patient developed paroxysmal palpitations, headaches, and hypertension during Kampo treatment. Notably, his tongue showed atypical signs of blood deficiency (pale white, thin, and emaciated appearance), despite other clinical findings suggesting sympathetic hyperactivity. From a Kampo medicine perspective, the patient exhibited a qi counterflow pattern and was treated with SRB, while his tongue signs were indicative of blood deficiency. Although SRB improved neuropsychiatric symptoms such as sleep disturbances, it did not affect hypertension or palpitations, ultimately leading to further investigation and the diagnosis of pheochromocytoma. The sympathetic hyperactivity symptoms observed in this case are characteristic manifestations of pheochromocytoma due to excessive catecholamine secretion. In Kampo medicine, such autonomic disturbances are understood through the concept of qi counterflow. Notably, the persistence of characteristic tongue signs, despite partial symptomatic improvement with SRB, provided an important clinical indicator that led to further investigation of underlying pathology. 

SRB, composed of 10 crude drugs, including Bupleurum Root, Pinellia Tuber, Cinnamon Bark, Poria Sclerotium, Scutellaria Root, Jujube, Ginseng, Oyster Shell, Longgu, and Ginger, has been studied for its effects on sympathetic activity. In animal studies, SRB has been shown to alleviate hyperexcitability and prolong sleep duration in insomnia models [11]. Experimental studies have also demonstrated that SRB reduces noradrenaline-induced vasoconstriction and hypertension in rabbits [12], while clinical studies have reported its effectiveness in treating various neuropsychiatric symptoms associated with autonomic dysfunction [13]. However, in our case, SRB only improved neuropsychiatric symptoms while having minimal impact on the underlying catecholamine excess caused by pheochromocytoma. This limited response to treatment suggested the presence of an organic cause beyond simple autonomic dysfunction.

**Figure 5:**
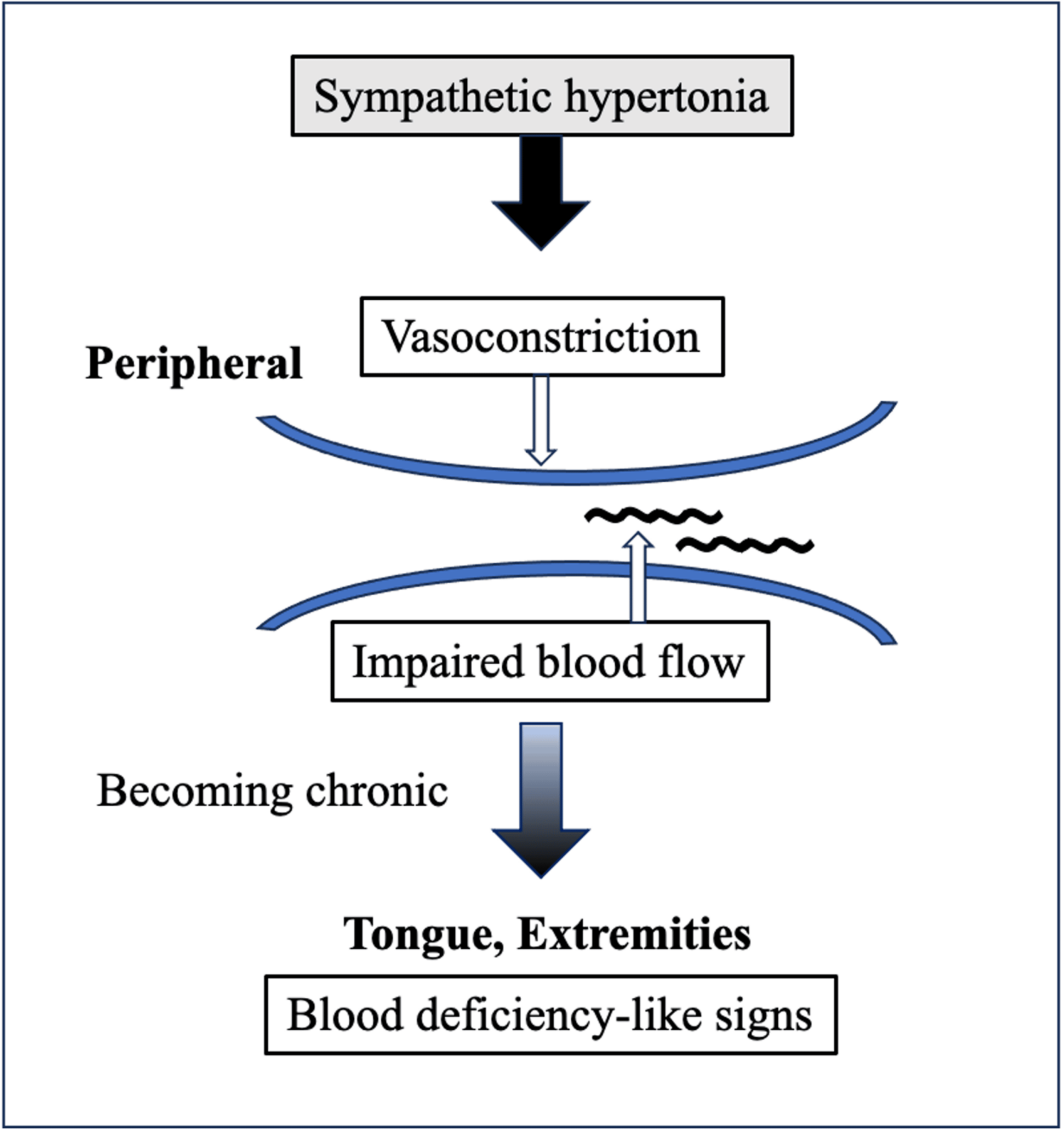
Suspected mechanism of the blood deficiency-like tongue signs. Sympathetic hypertonia causes peripheral vasoconstriction and impaired blood flow. When chronic, this leads to blood deficiency-like signs in the tongue and extremities. Image credit: Rinne Shimizu.

In cases of extreme sympathetic hypertonia, localized disruptions in blood flow can manifest as signs resembling blood deficiency, particularly in peripheral areas. The conventional diagnostic approach for sympathetic hyperactivity includes careful evaluation of cardiovascular symptoms, orthostatic changes, and autonomic function tests. Laboratory tests often include plasma catecholamines and thyroid function, as these conditions can present with similar autonomic symptoms. In our case, the patient's tongue turned from red to pale white following the onset of sympathetic hyperactivity. Furthermore, the tongue became noticeably thinner within a short period, showing these characteristic changes. We hypothesize that these tongue changes resulted from chronic peripheral vasoconstriction and impaired blood flow due to sustained sympathetic hypertonia (Figure 5). 

This peripheral circulatory disturbance, manifesting as both tongue signs (pale white, thin, and emaciated appearance) and cold extremities, serves as an important clinical indicator of systemic catecholamine excess effects on peripheral blood flow [14]. The combination of these blood deficiency-like tongue signs with other findings of peripheral vasoconstriction (qi counterflow) may help clinicians identify similar cases of sympathetic hyperactivity, which can be observed in various conditions such as hyperthyroidism, anxiety disorders, and drug-induced states (e.g., amphetamines, cocaine), as well as pheochromocytoma.

Pheochromocytoma is a neuroendocrine tumor primarily characterized by excessive catecholamine secretion [15]. This excessive catecholamine not only causes typical symptoms such as paroxysmal hypertension, hyperhidrosis, and headache but also induces significant peripheral vasoconstriction leading to a marked reduction in peripheral blood flow. Previous cases of pheochromocytoma have demonstrated that such vasoconstriction can lead to reversible cerebral vasoconstriction syndrome [16]. Another case of pheochromocytoma presented with a catecholaminergic crisis and profound lactic acidosis despite only mildly elevated blood pressure, suggesting that peripheral vasoconstriction can be more severe than indicated by blood pressure measurements [17]. These cases imply that peripheral vessels might be particularly susceptible to excessive catecholamine secretion from pheochromocytoma. The tongue is a highly vascularized organ that can be directly observed during physical examination. From the perspective of Kampo medicine, these changes in the tongue might reflect the intense peripheral vasoconstriction and subsequent tissue hypoperfusion caused by excessive catecholamines from pheochromocytoma.

To our knowledge, this is the first report describing tongue signs in a patient with pheochromocytoma. While the primary Kampo medicine findings pointed to qi counterflow, the tongue displayed signs of peripheral vasoconstriction. This discrepancy suggests that in conditions like pheochromocytoma, peripheral areas such as the tongue may exhibit signs of compromised blood flow due to localized blood flow issues. Given that catecholamine excess affects systemic circulation, similar tongue findings might be observed in other cases of pheochromocytoma. While Kampo's medical diagnoses can involve subjective elements, our observations raise interesting questions about the relationship between catecholamine excess and tongue manifestations that merit further investigation.

## Conclusions

This case report describes a patient with pheochromocytoma who presented with blood deficiency-like tongue signs. Our experience provides two important lessons. First, the interpretation of tongue signs requires consideration of autonomic nervous system activity, as sympathetic hyperactivity may produce signs that resemble blood deficiency. Second, integrating Kampo pattern identification with modern diagnostic methods can enhance clinical assessment. This integration of Kampo and Western diagnostic approaches can facilitate accurate diagnosis and optimize clinical outcomes.
